# Antifibrotic and Pro-regenerative Effects of SMAD3 siRNA and Collagen I mRNA-Loaded Lipid Nanoparticles in Human Tenocytes

**DOI:** 10.1021/acsanm.4c02996

**Published:** 2024-07-18

**Authors:** Sandra López-Cerdá, Giuseppina Molinaro, Rubén Pareja Tello, Alexandra Correia, Eero Waris, Jouni Hirvonen, Goncalo Barreto, Hélder A. Santos

**Affiliations:** † Drug Research Program, Division of Pharmaceutical Chemistry and Technology, Faculty of Pharmacy, University of Helsinki, 00014 Helsinki, Finland; ‡ Department of Hand Surgery, University of Helsinki and Helsinki University Hospital, 00029 Helsinki, Finland; ∇ Translational Immunology Research Program, Faculty of Medicine, University of Helsinki, PL 4 (Yliopistonkatu 3), 00014 Helsinki, Finland; ∥ Medical Ultrasonics Laboratory (MEDUSA), Department of Neuroscience and Biomedical Engineering, Aalto University, 02150 Espoo, Finland; ⊥ Orton Orthopedic Hospital, Tenholantie 10, 00280 Helsinki, Finland; # Department of Biomedical Engineering, The Personalized Medicine Research Institute (PRECISION), University Medical Center Groningen (UMCG), University of Groningen, Ant. Deusinglaan 1, 9713 AV Groningen, The Netherlands

**Keywords:** lipid nanoparticles, tendinopathy, siRNA, mRNA, regeneration

## Abstract

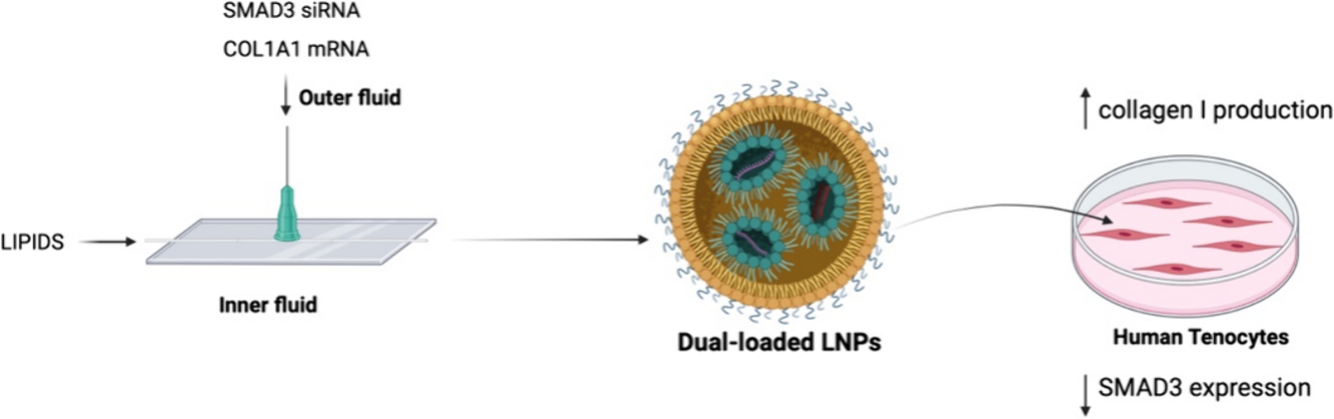

Tendinopathy involves the inflammation and degeneration of the tendon due to repetitive strain injury. Current treatments primarily target inflammation resolution, yet they do not aim at tissue regeneration. In this study, a microfluidics approach is harnessed to develop a platform of lipid nanoparticles (LNPs) loaded simultaneously with SMAD3 siRNA and collagen I mRNA, aiming to explore its potential dual antifibrotic and regenerative effects in human tenocytes. The developed LNPs displayed size homogeneity and colloidal stability and exhibited high cytocompatibility in human tenocytes. Moreover, LNPs allowed for efficient uptake and transfection efficiency of the RNAs. In the *in vitro* efficacy studies, the gene expression and production of SMAD3 and collagen I were tested by real-time quantitative chain polymerase reaction and immuno- and intracellular staining, revealing collagen I production enhancement, SMAD3 inhibition, and modulation of other tendon repair factors by the LNPs. Overall, the potential of this platform of RNA-loaded LNPs to be used as a dual therapeutic approach to prevent fibrosis and promote tissue remodeling in late stages of tendon diseases was confirmed.

## Introduction

Tendinopathy is a broad term that refers to all the inflammatory and degenerative processes that tendons undergo due to repetitive strain injury.^1,2^ Specifically, tendinitis denotates the inflammatory response that occurs in early stages of tendon injury, while tendinosis is related to tendon degeneration and fibrosis formation at late stages of tendon injury.^3−5^ Tendinopathies, affecting structures like Achilles tendon, patellar tendon, rotator cuff, and lateral and medial epicondyles, are significant causes of impairment worldwide, affecting both athletes and individuals of various age groups engaged in repetitive movements or who use strength at work.^6,7^ Consequently, tendinopathies impair daily activities and diminish quality of life, imposing substantial socioeconomic burden exciding EUR 180 billion in the USA and EU, with an increasing trend.^6^

For many years, the prevailing paradigm in the field of tendinopathy has been that inflammation is the primary aspect to be tackled, leading to research focused on developing drugs to resolve inflammation.^8,9^ Hence, the most commonly utilized therapeutics in the clinic for the management of tendinopathy include corticosteroids and nonsteroidal anti-inflammatory drugs (NSAIDs). These are often accompanied by physiotherapy and load modification, and in some cases, low-level laser ultrasound, extracorporeal shock wave therapy, and surgery are considered.^10−12^ However, these strategies have shown limited success since they fail to prevent the formation of fibrotic, scar tissue and do not promote the production and correct alignment of collagen I fibers, which should be favored over collagen III.^2,13^

Recently, strong evidence has emerged that inflammation is a necessary process in the first phase of a tendon repetitive strain injury. Furthermore, the primary aspect to address is the formation of scar tissue and an unorganized extracellular matrix (ECM) in the later stages of tendon repair. These factors hinder the complete healing of the tendon and lead to reinjuries.^9,14^ Several studies indicate that the strategy of administering growth factors, *e.g.,* transforming growth factor beta (TGF-β1), is undesirable since it leads to an excessive proliferation of tenocytes and ECM production.^14−16^ However, SMAD3 acts as a critical transcription factor for TGF-β1 and modulates the expression of genes involved in cell growth, inflammation and ECM formation.^17,18^ Therefore, SMAD3 expression is associated with the formation of scar tissue in healing tendons. There is supported evidence that impairment of SMAD3 signaling in tendinopathy could mitigate the pro-adhesion role of TGF-β1 without eliminating its other beneficial effects, thus making SMAD3 a potential therapeutic target.^17−20^

Moreover, type I collagen fibrils display high stiffness and confer mechanical strength and functionality to the tendon.^21,22^ In contrast, type III collagen fibrils, being thinner, form a randomly oriented network in the injury site during the healing process.^21−23^ Nevertheless, this asymmetric network must be gradually replaced by a stronger, better-aligned network of collagen I fibrils, since the ratio of collagen I to collagen III must be increased to promote tendon healing. Therefore, enhancing collagen I production can be beneficial at late stages of tendon disease.^23^ Hence, we hypothesize that a promising tendon tissue-specific therapeutic strategy could be based on silencing the expression of SMAD3 while simultaneously enhancing the production of collagen I.

The advances in the design and production of lipid nanoparticles (LNPs) for the encapsulation and delivery of RNAs, *i.e.*, small interfering RNA (siRNA) and messenger RNA (mRNA), have led to the clinical translation of a novel therapeutic to treat transthyretin amyloidosis and of novel vaccines for preventing viral infection.^24−26^ While delivering an siRNA allows silencing the expression of a deleterious gene, delivering an mRNA enhances the production of a therapeutic one, giving an opportunity for the development of tissue- or even cell-specific treatments with less collateral effects and improved therapeutic outcomes.^27,28^ One of the reasons why LNPs are the most successful platform to deliver RNAs is that the chemical structure of the cationic and ionizable lipids used in these formulations has been specifically fine-tuned to encapsulate these sensitive biomolecules, deliver them inside cells and disrupt the endosomal compartment to release them to the cytosol.^29,30^ In addition, the advances in the microfluidics technology have allowed development of fast and reproducible methods to produce LNPs, accelerating the development of new lipid-based nanoplatforms for different therapeutic applications.^31−33^

**Figure 1:**
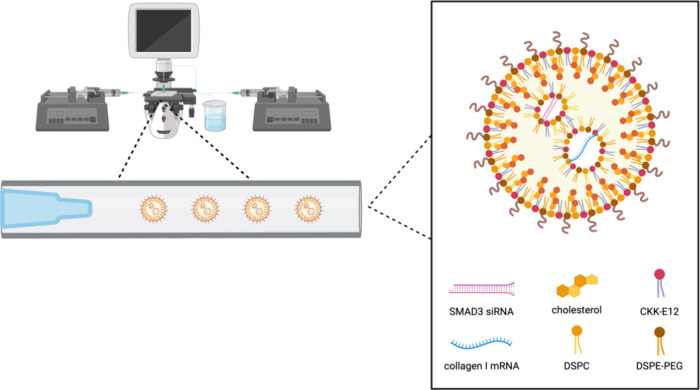
Schematic illustration of the one-step microfluidics coflow nanoprecipitation method developed to produce LNPs loaded simultaneously with SMAD3 siRNA and collagen I mRNA. Image created with Biorender.com.

In this study, we propose the design, optimization, and development of a nanoplatform of siRNA and mRNA-loaded LNPs as well as the *in vitro* testing in human tenocytes.^34,35^ Specifically, lipid nanoparticles were loaded with SMAD3 siRNA and collagen (COL1A1) mRNA with the aim of developing a therapeutic strategy that combines fibrosis prevention and remodeling into a collagen I-rich ECM, respectively. The design of the nanoparticles was inspired in our previous works and by others,^35−37^ while the production was performed by a newly optimized microfluidics coflow method, which allows the reproducibility and potential up-scalable production of particles (Figure 1). The size, size homogeneity, and morphology of this formulation were evaluated by using dynamic light scattering and transmission electron microscopy, and the encapsulation of the payloads was confirmed by the Ribogreen assay. For the *in vitro* studies, human tenocytes isolated from human injured tendons were used, and the cytocompatibility and cell uptake studies were performed to assess the cell-nanoparticle interactions. The potential therapeutic efficacy of this platform of siRNA and mRNA-loaded LNPs was confirmed by *in vitro* studies involving cell and molecular biology techniques along with immunofluorescence studies confirming the modulation of the expression of the targets at gene and protein levels.

The overall aim of this work was to develop a nanoparticle-based platform for the dual delivery of a relevant mRNA and siRNA with potential therapeutic application in preventing fibrosis and promoting tendon remodeling during the late stages of tendon tissue healing.

## Results and Discussion

### Physicochemical Characterization and Colloidal Stability of LNPs

LNPs, SMAD3 siRNA, and collagen I (COL1A1) mRNA single loaded-LNPs as well as SMAD3 siRNA and COL1A1 mRNA dual-loaded LNPs were fabricated using microfluidics, employing an already optimized coflow glass capillary device.^38,39^ Briefly, cKK-E12, DSPC, cholesterol, and DSPE-PEG (at the molar ratio of 50:10:38.5:1.5) were dissolved in the ethanolic organic phase, which was injected in coflow with an aqueous phase of 1% (poly(vinyl alcohol)) PVA in RNase-free Milli-Q water.^36^ The flow rate ratio of the aqueous and organic phases and the concentration of lipids in the organic phase were modified systematically for optimization. Flow rates of 1 to 20 mL/min for the organic phase to the aqueous phase, respectively, and a concentration of organic phase of 5 mg/mL, were selected as optimal (Table S1). After purifying LNPs by dialyzing overnight against an excess of Milli-Q water, empty LNPs with a size of 151 nm, polydispersity index (PDI) of 0.3, and zeta-potential of almost +30 mV were obtained, as measured by dynamic light scattering (DLS) and electrophoretic light scattering (ELS). Therefore, the same formulation and process parameters were translated to produce the single-loaded with SMAD3 siRNA and COL1A1 mRNA, and with the dual-loaded LNPs. For all the RNA-loaded LNPs, a weight ratio of 12.5:1 between the cationic lipid cKK-E12 and the corresponding RNA was selected for allowing RNA complexation, based on previous optimizations.^36^

**Figure 2:**
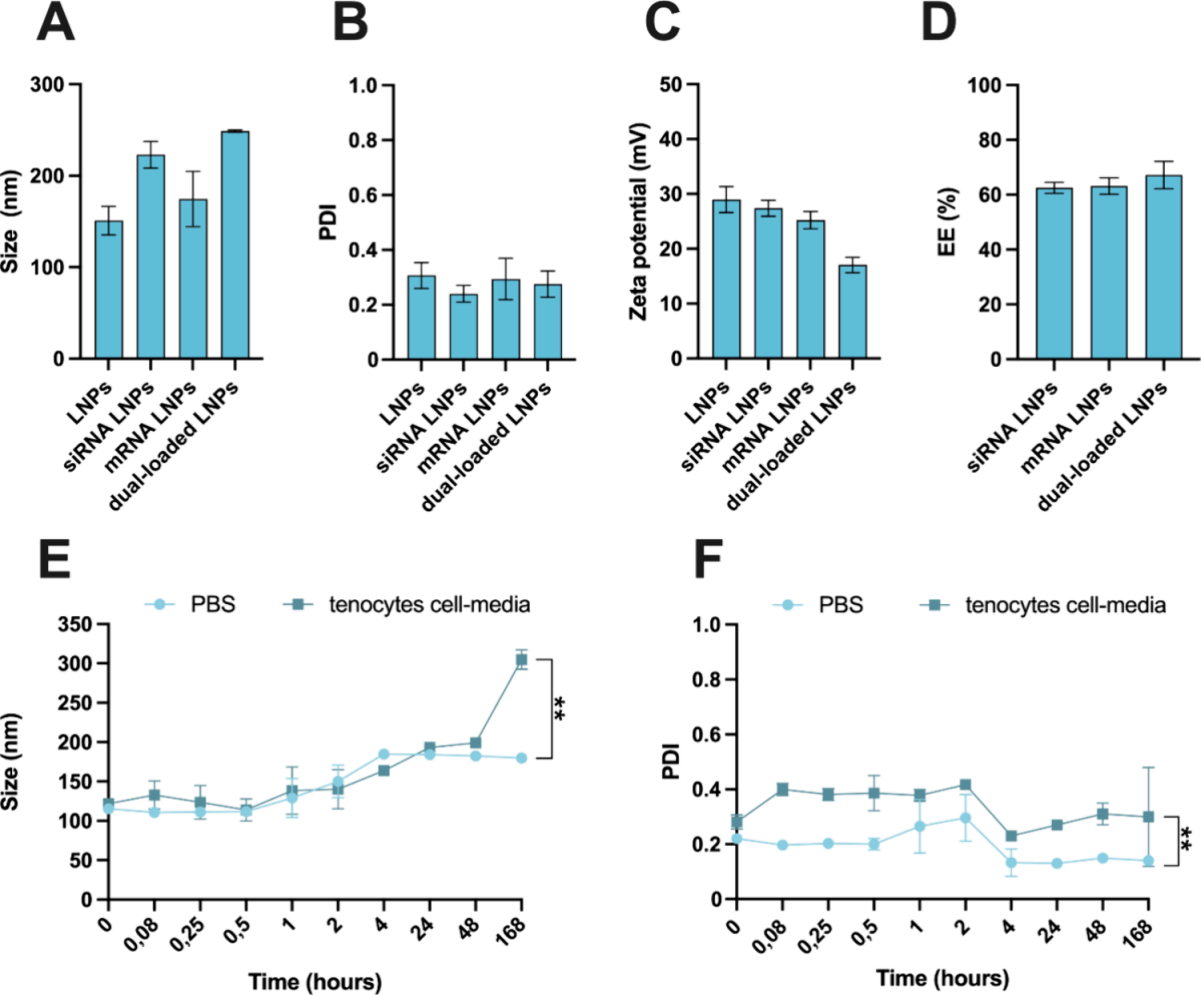
Physicochemical characterization of the optimized empty LNPs, SMAD3 siRNA LNPs, COL1A1 mRNA LNPs, and dual-loaded LNPs regarding the (A) size (nanometers), (B) PDI, and (C) zeta potential (mV), and EE (D). Colloidal stability of empty LNPs in tenocytes’ cell culture medium and 1× PBS in terms of (E) size and (F) PDI. All the samples were analyzed after dialysis and the results are presented as mean ± s.d. (*n* ≥ 3). For statistical analysis, a paired Student’s *t*-test was used, and *p*-values were set at probability **p < 0.01.

In Figure 2A, the size of siRNA LNPs, mRNA LNPs, and especially dual-loaded LNPs was higher than that of the empty LNPs, being ∼250 nm for the dual-loaded LNPs. However, the PDI of all RNA-loaded LNPs was practically the same as that of empty LNPs, confirming size homogeneity despite the single and dual loading of siRNA and mRNA payloads. The zeta-potential (Figure 2C) decreased to +18 mV in the case of the dual-loaded LNPs, which can be a proof that part of the RNA chains are on the surface or partially complexed by the lipids.^40^ Overall, these LNPs proved suitable size and size homogeneity for local delivery, which is the most desirable delivery route for the treatment of soft tissue injuries like tendinopathy.^41^ In addition, the overall positive zeta-potential is also desirable for long-term particle stability and efficient RNA encapsulation. According to the differential quantification of the amount of total RNA in the LNPs minus the RNA quantified outside of the LNPs using the standardized Ribogreen assay, siRNA LNPs and mRNA LNPs displayed an encapsulation efficiency (EE) of ∼63% (Figure 2D). Similarly, dual-loaded LNPs displayed an EE of siRNA + mRNA of 67% (Figure 2D), which confirms the successful encapsulation of the payloads inside the LNPs and its protection from degradation.

The colloidal stability of the developed LNPs was also investigated in phosphate buffer saline solution 1× (PBS) and in human tenocytes cell culture media, considering that all the *in vitro* studies were conducted on tenocytes. When particles were tested in both cell media and PBS, there was an increase of the size over the time (Figure 2E). Additionally, the PDI was increased in cell culture medium more than in PBS at all time points tested (Figure 2F). The higher size heterogeneity in cell culture medium is a phenomenon previously reported and is justified by the fact that the serum of the cell culture medium contains proteins, which can interact with the surface of nanoparticles, establishing surface interactions and forming nanoparticle–protein complexes or a protein corona around the particles.^42,43^ Nevertheless, LNPs were not aggregating in cell culture medium at any of the time points tested, proving its suitability for the later *in vitro* testing.

**Figure 3:**
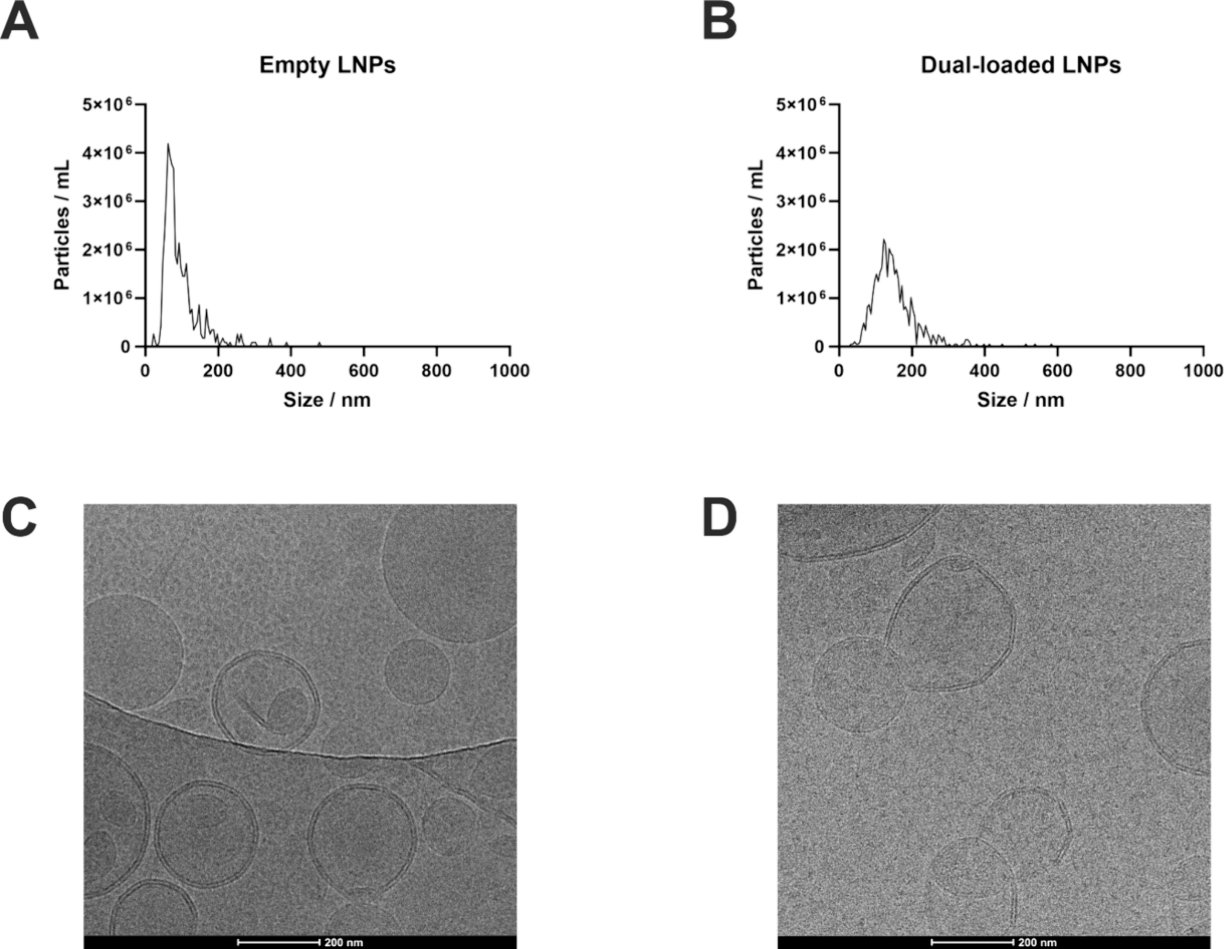
Size distribution and concentration of empty LNPs (A) and dual-loaded LNPs (B) as measured by NTA (samples diluted 10,000 times and 5,000 times, respectively). Cryo-EM images display the morphological structure and size homogeneity of empty LNPs (C) and dual-loaded LNPs (D). Scale bars are 200 nm in each image.

Next, cryogenic electron microscopy (Cryo-EM) analysis and nanoparticle tracking analysis (NTA) were conducted in empty LNPs and dual-loaded LNPs to contrast the size and size homogeneity data obtained with DLS and to also determine the particles’ concentration and morphology. On the one hand, in Figure 3A,B, empty and dual-loaded LNPs displayed average sizes of 120 and 220 nm, respectively, which are similar to those obtained by DLS (Figure 2A). In addition, the particle concentration was of 4.5 × 10^11^ million particles per mL and 1.0 × 10^10^ particles per mL for empty LNPs and dual-loaded LNPs, respectively (Figure 3A,B), taking into account the dilution factors. This confirms that an acceptable nanoparticle throughput was obtained even if a low amount of lipids was injected against a large volume of aqueous phase in the optimized microfluidics method. On the other hand, the Cryo-EM data confirmed that particles were spherical in shape and displayed the typical appearance of LNPs reported by others (Figure 3C,D).^44,45^ The size homogeneity of the empty and dual-loaded LNPs is in accordance with the size and PDI data obtained by DLS, since some particles are above and others below the average size measured by DLS. This confirmed that the empty and dual-loaded LNPs displayed acceptable size homogeneity for local delivery applications. Therefore, it was verified that the microfluidics production method and the formulation components used in this formulation, especially the poly(ethylene) glycol (PEG) used as the stabilizer, ensure the stability of the particles and prevent their potential aggregation.^46^

### Cell Viability Studies

**Figure 4:**
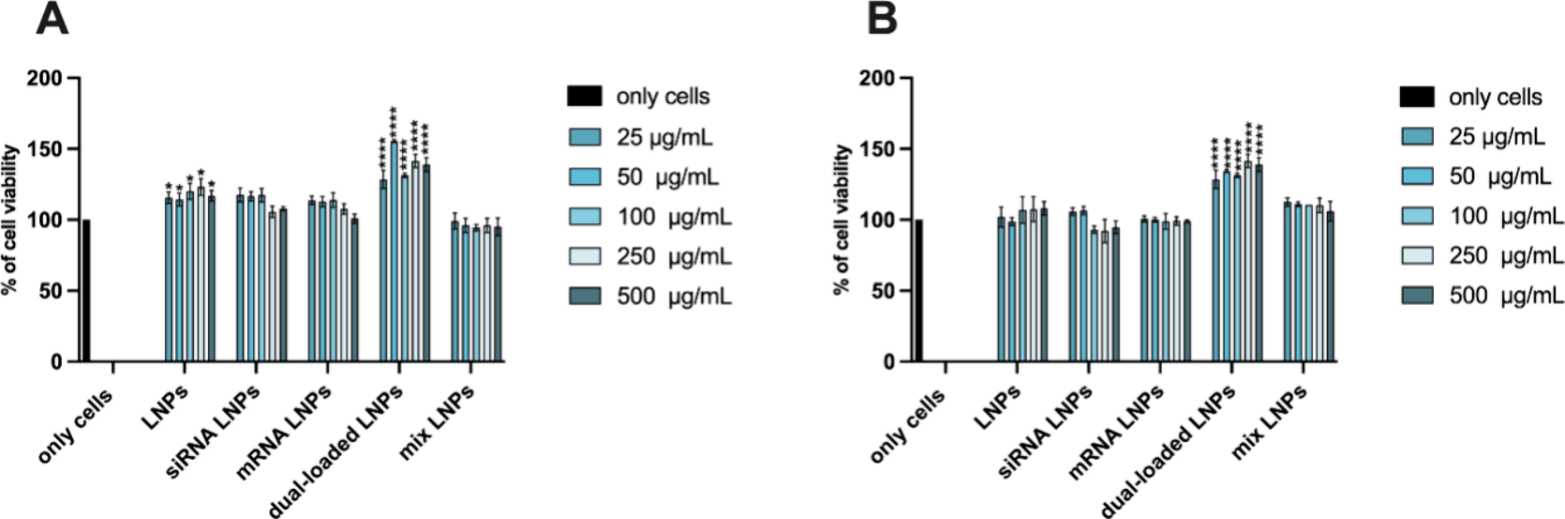
Cell viability studies of LNPs, siRNA-loaded LNPs, mRNA-loaded LNPs, dual-loaded LNPs, and mixed LNPs on human tenocytes after (A) 24 h and (B) 48 h. Cell culture media represented negative positive control. Results are presented as mean ± SD (*n* ≥ 3). For statistical analysis, a one-way ANOVA followed by a Dunnett posthoc test was used. The probabilities were set at *p < 0.05, ***p < 0.001, and ****p < 0.0001 for comparing all the samples vs only cells.

The cell viability of SMAD3 siRNA LNPs, COL1A1 mRNA LNPs, dual-loaded LNPs, and mix LNPs was assessed in human primary tenocytes to confirm their safety profile *in vitro* to establish a safe LNP concentration for further testing. The cell viability was assessed using the CellTiter-Glo luminescence assay, which quantifies the production of adenosine triphosphate (ATP)-luciferase.^47^ Based on previous studies by our lab,^36,37^ the concentrations used were 25, 50, 100, 250, and 500 μg/mL and the incubation times of 24 h (Figure 4A) and 48 h (Figure 4B) were chosen according to the incubation time for transfection efficiency and the data from previous studies.^36,37,48^ Tenocytes in cell medium were used as a negative control for establishing the 100% cell viability reference value. As shown in Figure 4A,B, the different formulations have a good cell viability, demonstrated by the absence of toxicity up to the highest concentration of 500 μg/mL. A certain increase in the cell viability over 100% was observed when human tenocytes were treated with dual-loaded LNPs. This phenomena has been observed before and can be attributed to the interactions of the assay with the LNPs and the variability of the assay.^48^

### Transfection Efficiency

**Figure 5:**
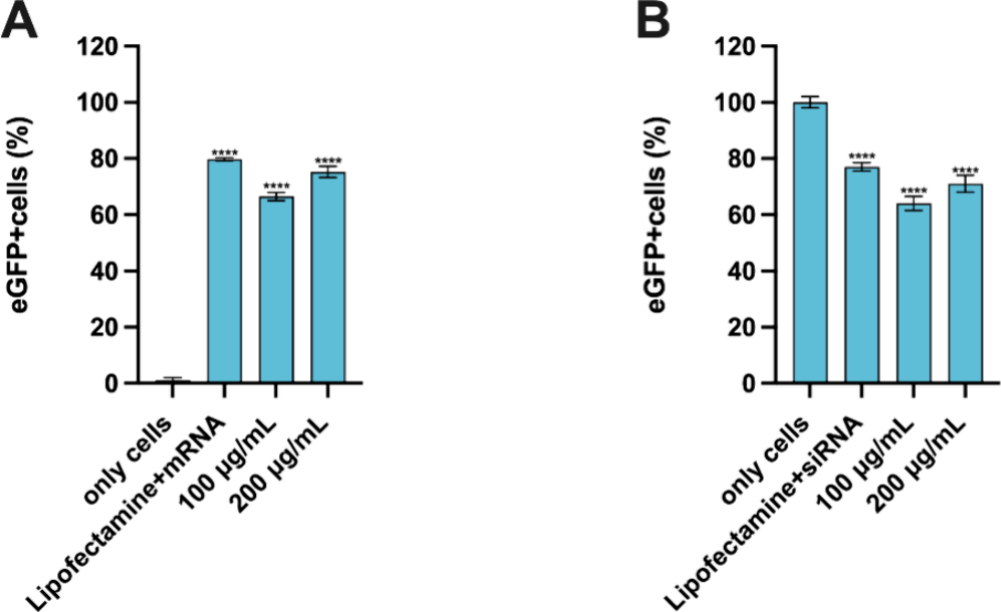
(A) Flow cytometry study of the transfection efficiency of mRNA LNPs in RAW 264.7 cells. (B) Flow cytometry study of the transfection efficiency of siRNA LNPs in eGFP-expressing RAW 264.7 cells. Results are represented as percentage of eGFP^+^ cells ± s.d. (*n* = 3). Results are presented as mean ± SD (*n* ≥ 3). For statistical analysis, an ordinary one-way ANOVA followed by a Dunnett posthoc test was used. The probabilities were set at ****p < 0.0001, comparing all the samples vs only cells.

Next, the ability of this formulation to allow for functional RNA delivery was evaluated. For this, the efficiency of these LNPs in enabling particle uptake and endosomal escape of the mRNA and siRNA payloads, leading to gene expression and silencing, respectively, was tested. RAW 264.7 cells and eGFP-expressing RAW 264.7 cells were used, since they are common *in vitro* models for evaluating the transfection efficiency of LNPs in early stages of formulation development,^49^ and there is not any already established model of eGFP-expressing tenocytes. Specifically, RAW 264.7 cells were used for assessing the transfection efficiency of LNPs loaded with the model eGFP-mRNA, and eGFP-expressing RAW 264.7 cells were used for assessing the transfection efficiency of LNPs loaded with the model eGFP-siRNA. Figure 5A confirms that LNP concentrations of 100 μg/mL led to an increase of the expression of eGFP of ∼80% compared to the only cell control, similar to the lipofectamine positive control. Furthermore, concentrations of LNPs of 100 μg/mL led to almost 40% silencing of eGFP in eGFP-expressing RAW 264.7 cells, displaying an efficiency higher than that of the positive control of lipofectamine + eGFP siRNA (Figure 5B). A further confirmation of the siRNA transfection efficiency is that when loading serpine1 siRNA (irrelevant gene in this study) in these LNPs, the expression of this gene was significantly downregulated with respect to the only cells control (Figure S1). Hence, these results support the suitability of this formulation of LNPs for functional delivery of siRNAs and mRNAs. Overall, based on the cell viability results and the transfection efficiency study, a concentration of LNPs of 100 μg/mL (which is equivalent to 0.3 μg/mL of the corresponding RNAs) was selected for the next *in vitro* mechanistic and efficacy studies as a safe concentration that can potentially allow for therapeutic efficacy.

### Quantitative and Qualitative Cell Uptake

The interaction between LNPs and human tenocytes was evaluated quantitatively and qualitatively in human tenocytes. For the cell uptake study, LNPs were prepared using fluorescein isothiocyanate (FITC)-labeled PEG, which emits fluorescence when excited by a 488 nm laser. LNPs were used at a concentration of 100 μg/mL (equivalent to 0.3 μg/mL) in both studies.

**Figure 6:**
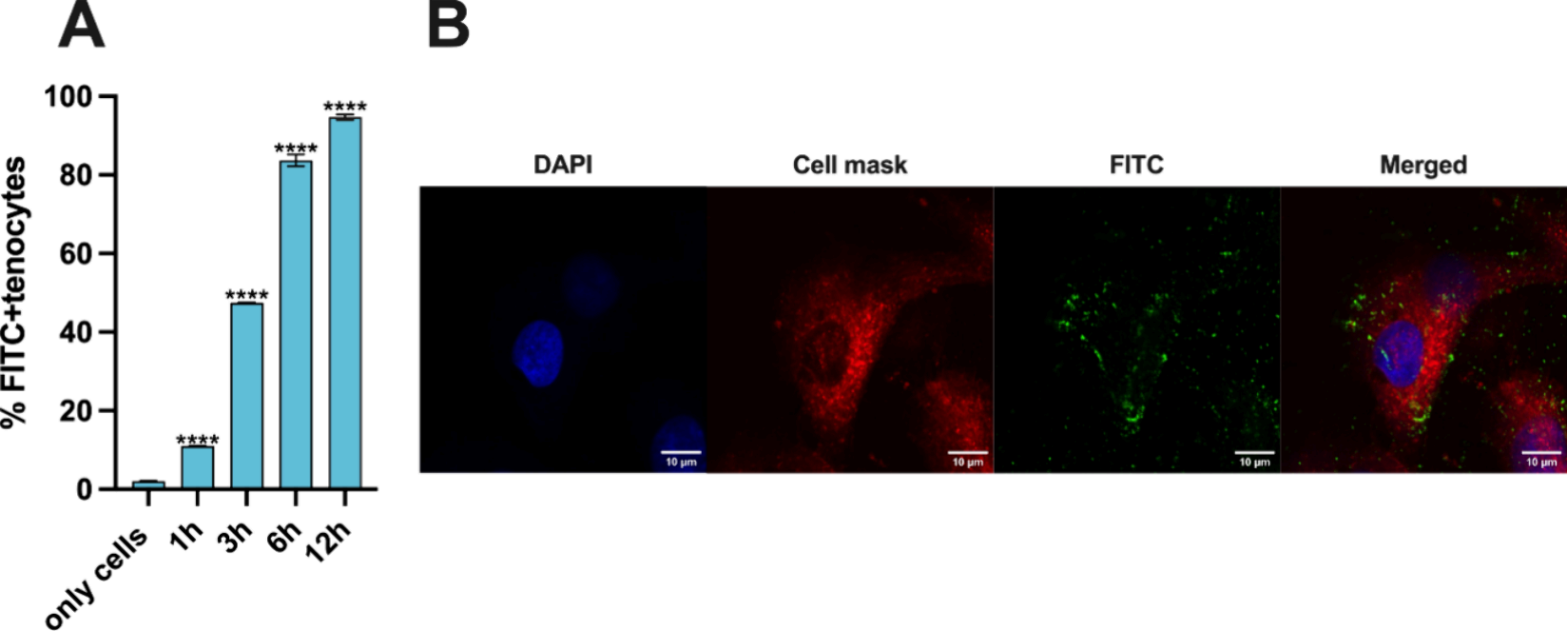
(A) Quantitative cell uptake study on human primary tenocytes using a flow cytometer. Cells were incubated for 1, 3, 6, and 12 h with 50 μg/mL of FITC-labeled LNPs. Results are presented as mean ± SD (*n* ≥ 3). For statistical analysis, a one-way ANOVA followed by a Dunnett posthoc test was done. The probabilities were set at ****p < 0.0001, comparing in all the samples vs only cells. (B) Qualitative uptake studies of LNPs in human tenocytes performed by confocal microscopy. The cell uptake was evaluated by confocal fluorescence microscopy after incubation with the FITC-labeled LNPs for 6h at 37 °C. The green channel corresponds to FITC (NPs), the blue channel corresponds to DAPI (nuclei), and the red channel corresponds to CellMask (cell membrane). Scale bars are shown in each image.

For quantitative uptake studies, the FITC-labeled LNPs were incubated with tenocytes for 1, 3, 6, and 12 h, and then cells were collected to be analyzed by flow cytometry. As shown in Figure 6A, a time-dependent uptake can be observed, where more than 80% of the FITC-labeled LNPs are internalized after 6 h and the uptake starts to stabilize from 6 h up to 12 h, when the maximum of 90% of uptake is reached. The low LNP uptake at the low time point of 1 h is in accordance with the colloidal stability of the LNPs demonstrated in the colloidal stability study in tenocytes’ media (Figure 2D,E), which confirms that LNPs remain in suspension in the cell medium without aggregating, and therefore it takes time for them to interact with the adherent tenocytes. In spite of this, the positive charge of the LNPs aids in the electrostatic interaction with the negatively charged cell membrane, allowing for efficient cell internalization after higher time points.^50^

The qualitative cell uptake was also evaluated in human tenocytes by imaging the samples with confocal microscopy after 6 h of incubation with the FITC-labeled LNPs. One single cell was selected from a population to better visualize the internalization of the particles. Figure 6B shows that the FITC-labeled LNPs are internalized by the cells, further supporting the observation of the quantitative uptake analysis. Confocal images showing larger cell populations are shown in Figure S2 and further support the previously mentioned observations.

### Modulation of the Expression of Pro-fibrotic and Tendon-Remodeling Genes by RNA LNPs

The TGF-β/Smad2/3 signaling pathway is considered crucial for regulating the formation of fibrotic tissue in injured tendons.^51^ Previous studies have demonstrated that knocking down SMAD3 can promote scarless tendon remodeling in late stages of tendon repair by decreasing the levels of collagen III, modulating matrix anabolism and minimizing the proliferation, migration, and differentiation of tenogenic stem cells.^17,52,53^ Bone morphogenetic protein 2 (BMP-2) is a mediator in the TGF-β/Smad2/3 signaling pathway and plays an important role in ECM synthesis and tendon healing.^54^ In previous studies, the possibility of enhancing BMP-2 for tendon healing and fibrosis prevention has been acknowledged.^55,56^ Hence, the antifibrotic effect of SMAD3 siRNA and the pro-regenerative effect of COL1A1 mRNA were evaluated by evaluating the gene expression of *SMAD3, BMP2, COL3A1*, and *TGFB1*, using the real-time quantitative polymerase chain reaction (RT-qPCR).

Human tenocytes were treated with siRNA LNPs, mRNA LNPs, dual-loaded LNPs (LNPs concentration of 100 μg/mL equivalent to 0.3 μg/mL of siRNA and mRNA, respectively), mix LNPs (100 μg/mL of siRNA LNPs + 100 μg/mL of mRNA LNPs equivalent to siRNA and mRNA concentrations of 0.3 μg/mL, respectively) and the controls SMAD3 siRNA, COL1A1 mRNA, empty LNPs, and DMEM-F12 medium, for 48 h.^36,37^

**Figure 7:**
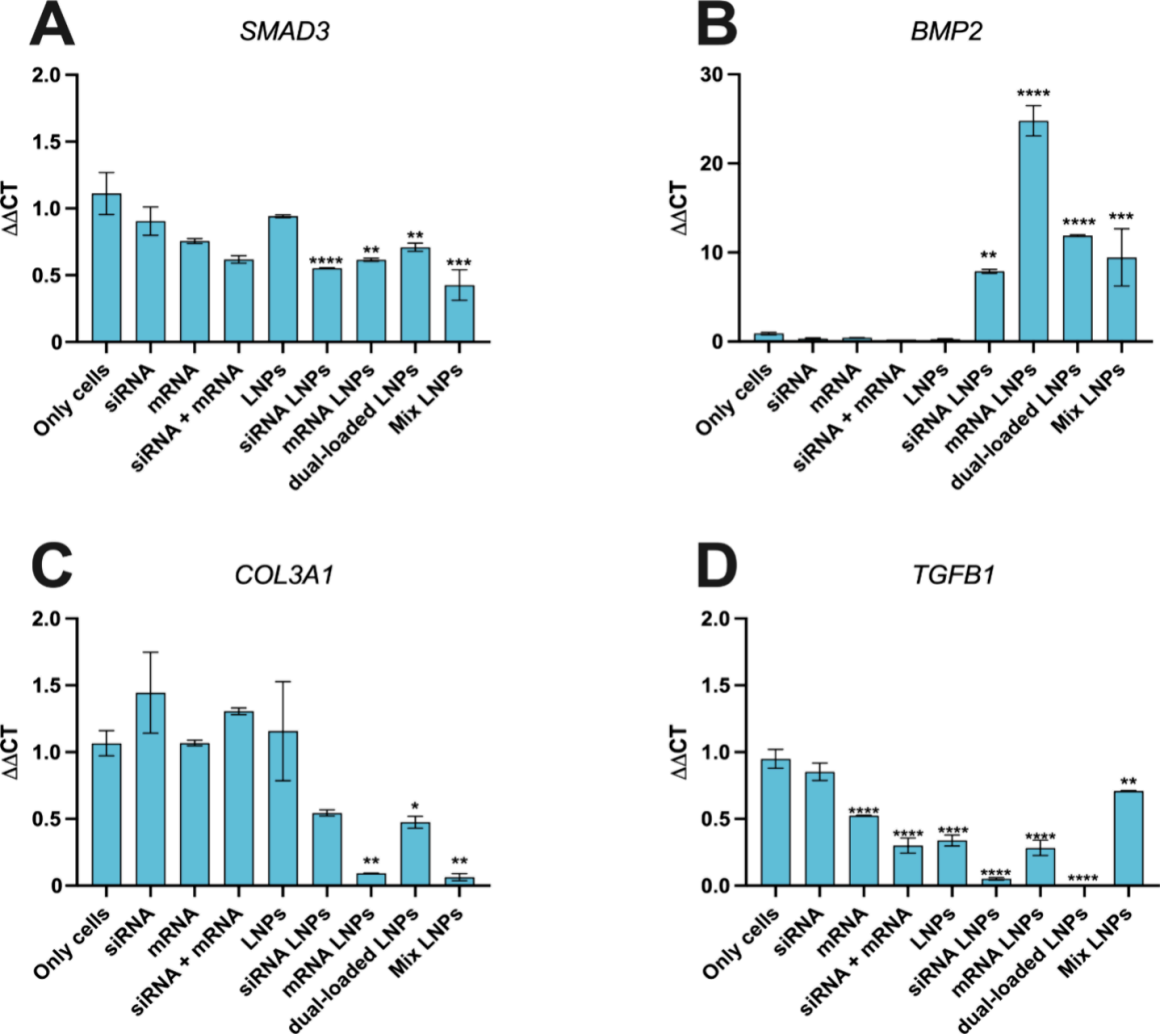
RT-qPCR study to assess the gene expression of (A) *SMAD3*, (B) *BMP2*, (C) *COL3A1*, and (D) *TGFB1* in human tenocytes. Data show the fold increase values compared to the only cells control ± s.d. (*n* ≥ 3). For statistical analysis, an ordinary one-way ANOVA followed by a Dunnett posthoc test was used. The significance levels were set at the probabilities of *p < 0.05, **p < 0.01, ***p < 0.001, and ****p < 0.0001 for comparison with only cells (negative control).

In Figure 7A, the gene expression of SMAD3 was significantly downregulated by the siRNA LNPs, mRNA LNPs, dual-loaded LNPs, and mix LNPs by almost 2-fold with respect to the control of only cells. The silencing of SMAD3 in the LNP-treated samples as compared to the nontreated cells is a relevant finding because the tenocytes used for this work are extracted from patients with late-stage tendinopathy that underwent surgery when the tendon tissue displays a pro-fibrotic profile, meaning that the cells in the control are presumably pro-fibrotic. Interestingly, the mRNA LNPs also reduced the expression of SMAD3, but the dual-loaded LNPs did not reduce the SMAD3 expression further than the siRNA LNPs, which suggests that the expression of this gene cannot be completely silenced since there are many other factors that can activate it simultaneously.^19,56,57^ The BMP-2 expression was significantly enhanced when the cells were treated with the siRNA LNPs, mRNA LNPs, dual-loaded LNPs, and mixed LNPs (Figure 7B). The interplay between BMP-2 and SMAD3 can be bidirectional, and many studies report that BMP-2 can promote Smad and non-Smad signaling routes parallelly, which can justify that SMAD3 can be simultaneously activated by BMP-2.^56^ The observed BMP-2 expression enhancement can be due to the synergistic effects of the SMAD3 siRNA and COL1A1 mRNA and is actually a beneficial side effect of this therapeutic approach, since BMP2 enhancement has been considered as a possible therapeutic approach for preventing fibrosis and promoting tissue remodeling.^58,59^ In line with the SMAD3 silencing, the expression of COL3A1 was downregulated by the siRNA LNPs, mRNA LNPs, dual-loaded LNPs, and mix LNPs (Figure 7C). This is in accordance with the forecasted effects of SMAD3 reducing the production of collagen and is beneficial to promote a higher col I to col III ratio and favor the symmetric entanglement of the ECM.^58,60^ Finally, the expression of TGF-β was downregulated when the cells were treated with the control RNAs but was even more evident when the cells were treated with the siRNA LNPs and dual-loaded LNPs (Figure 7D). This further demonstrates that SMAD3 silencing was contributing to downregulation of the main fibrotic markers.^17^

Overall, the effect of SMAD3 siRNA delivered by LNPs at the gene level on human tenocytes was confirmed. Some synergistic effects of SMAD3 siRNA and COL1A1 delivered by LNPs were also observed and a complex interplay between the factors interfering in the TGF-β/Smad2/3 signaling pathway was identified, in agreement with previous studies.^18,55,56^

### Modulation of the Production of Collagen I and Smad3 by RNA LNPs

The modulation of the gene expression levels do not always correspond to the modulation of the expression at the protein level since protein translation occurs after RNA transcription.^61^ Therefore, the extracellular production of COL1A1 and the intracellular production of SMAD3 were evaluated by performing immunostaining and intracellular staining assays, respectively.

**Figure 8:**
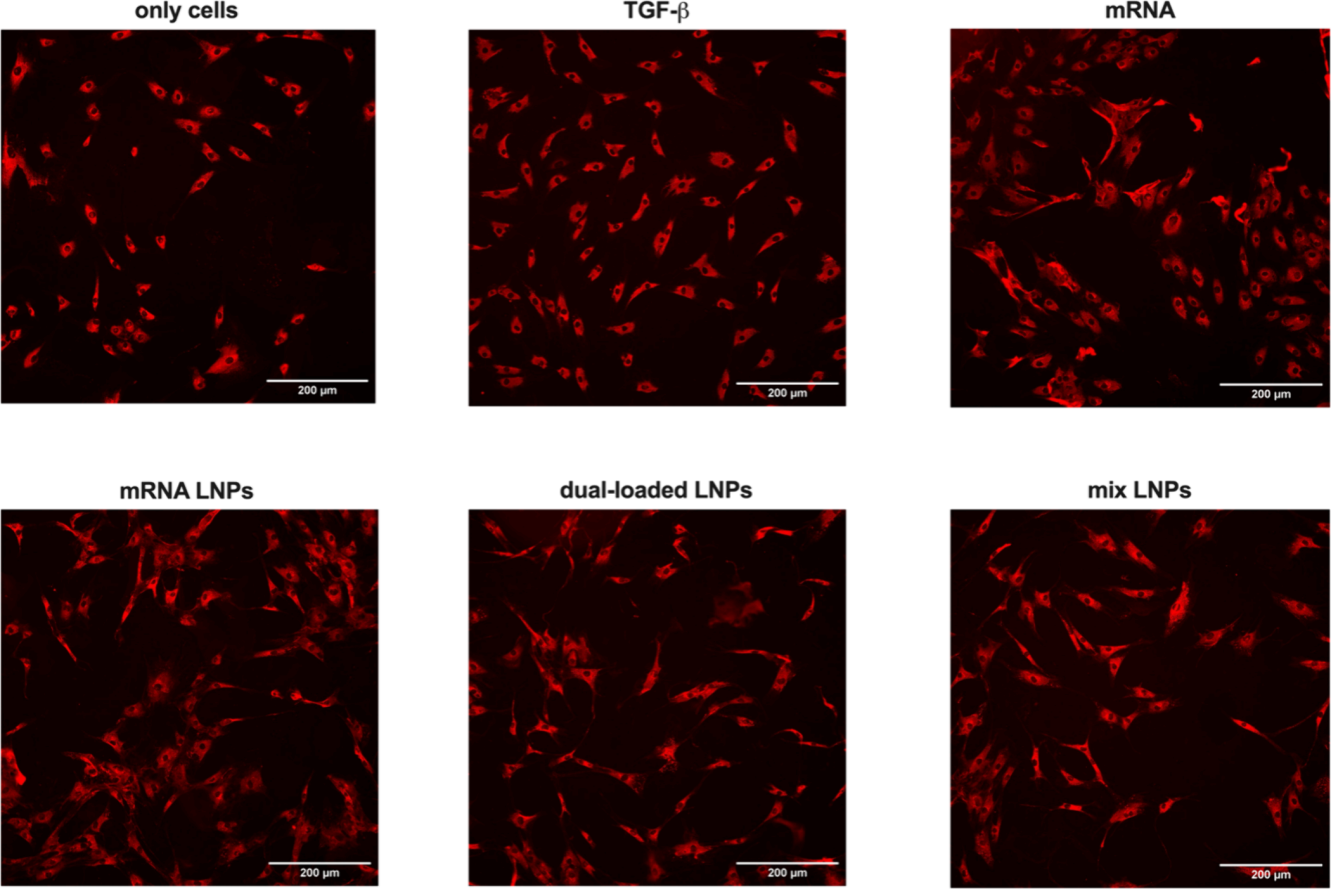
Representative images of the immunofluorescence staining of collagen I in a monolayer culture of human tenocytes visualized by a MolecularDevices Nano Instrument. Collagen I was quantified 48 h after the treatment with COL1A1 mRNA, mRNA LNPs, dual-loaded LNPs, and mix LNPs using an anticollagen type I antibody. Cells treated with TGF-β (20 ng/mL) were used as a positive control. 32 images were taken per well.

Figure 8 shows representative images of collagen I immunostaining. It can be seen that tenocytes treated with mRNA LNPs, dual-loaded LNPs, and mix LNPs start to produce collagen fibers around the cell cytoplasm at higher extent than in nontreated tenocytes and tenocytes treated with COL1A1 mRNA alone.

**Figure 9:**
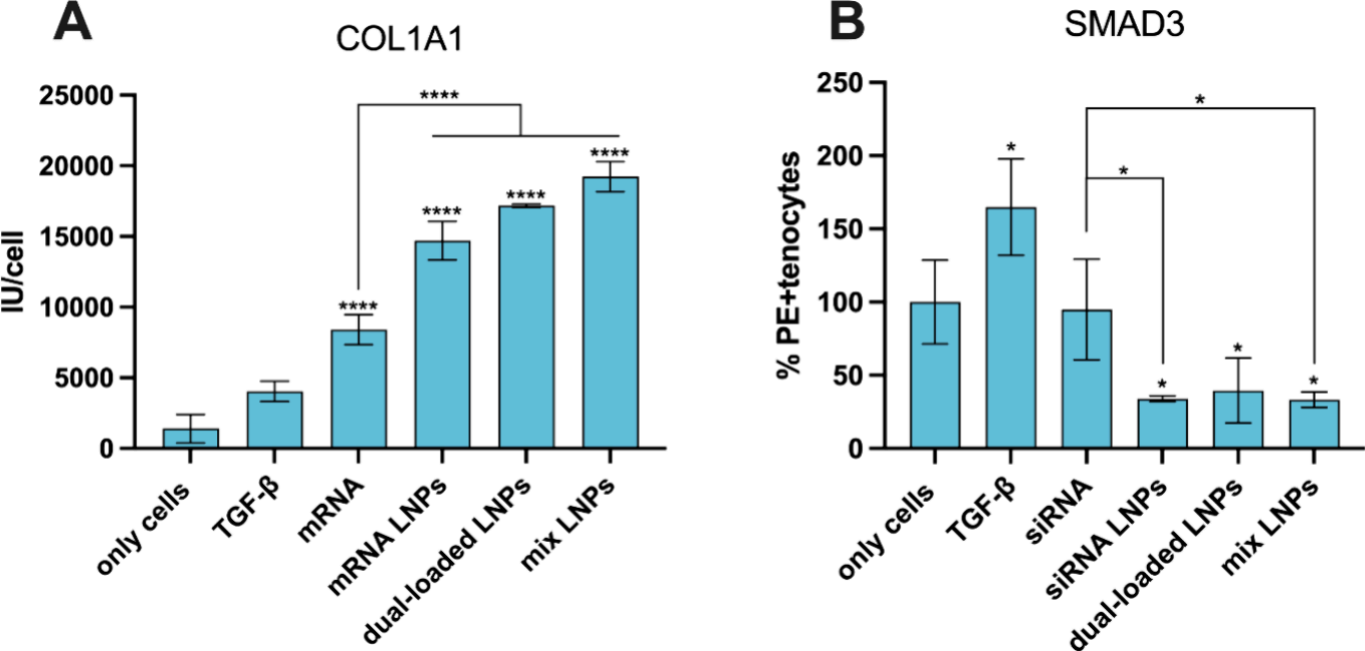
(A) Quantitative analysis of collagen I production using MetaXpress software. The results represent the sum of the intensity values of Texas Red divided by the count of cells. The 32 images were analyzed per well and per sample. (B) Flow cytometry analysis of SMAD3 expression after intracellular staining. For statistical analysis, a one-way ANOVA followed by a Dunnett posthoc test was used in (A) and (B). The significance levels were set at the probabilities of *p < 0.05, and ****p < 0.0001 for comparison with only cells (A, B), mRNA (A), and siRNA (B).

The qualitative observations can be better justified by the quantitative analysis of collagen I production shown in Figure 9A. As can be seen, the production of collagen I is enhanced significantly when tenocytes are treated with the mRNA LNPs, dual-loaded LNPs, and mix LNPs, and this enhancement is statistically significant when compared to mRNA alone, proving the benefit of using the LNP delivery system. In addition, the enhancement exerted by the mRNA and the mRNA-containing LNPs is statistically significant when compared to the TGF-β positive control. Furthermore, it should be noted that the enhancement of collagen I production by the dual-loaded and mix LNPs demonstrated that delivering COL1A1 mRNA can compensate for the inhibitory effects of SMAD3 silencing in collagen production, which justifies the combination of RNA therapeutics chosen in this study.^58,60^ Moreover, Figure 9B shows that the intracellular production of SMAD3 is decreased by the siRNA LNPs, dual-loaded LNPs, and mixed LNPs in comparison with the control of cells alone, while the siRNA alone did not decrease the production of SMAD3 compared to the control, proving the relevant role of LNPs to achieve a functional delivery of the RNAs.

Overall, the efficient delivery of the COL1A1 mRNA and the SMAD3 siRNA by both coloaded and mixed LNPs was confirmed also at the protein level and proves the therapeutic efficacy of this nanoplatform in human tenocytes *in vitro*.

## Conclusions

Here, a new LNP platform for simultaneous delivery of SMAD3 siRNA and COL1A1 mRNA was developed by using microfluidics technology as a potential therapeutic approach for treating tendinopathy. The LNPs were homogeneous in size and stable in different media. Moreover, the nanosystem demonstrated a good cytocompatibility profile in human tenocytes up to the highest concentration of 500 μg/mL. The LNPs showed high internalization in human tenocytes, reaching a maximum at 12 h after incubation. The *in vitro* efficacy studies confirmed the downregulation of SMAD3 and the upregulation of collagen I production, respectively, as well as the modulation of other fibrosis-related and tendon-remodeling factors. In addition, the complex interplay between the factors interfering in the TGF-β/Smad2/3 signaling pathway was confirmed upon LNP treatment. Thus, our study highlights the potential therapeutic benefits of developing combinatorial therapeutic approaches for targeting complex diseases like tendinopathy. Overall, this nanoplatform of RNA-loaded LNPs is proposed as a promising candidate for further development of a dual therapeutic approach to simultaneously prevent fibrosis and promote tissue remodeling in the late stages of tendon diseases.

## Materials and Methods

### Materials for LNP Preparation

cKK-E12 was purchased from Echelon Bioscience (Salt Lake City, Utah). 1,2-Distearoyl-*sn*-glycero-3-phosphocholine (DSPC) and cholesterol were obtained from Avanti Polar Lipids (Alabaster, AL, USA). 1,2-Distearoyl-*sn*-glycero-3 phosphoethanolamine-*N*-[carboxy(polyethylene glycol)-2000] (DSPE-PEG) was purchased from Merck (Germany). DSPE-PEG-FITC was purchased from Nanosoft Polymers.

eGFP siRNA and SMAD3 siRNA were obtained from Eurogentec (Seraing, Belgium). eGFP and COL1A1 mRNA were purchased from RiboPro (The Netherlands). Diethyl pyrocarbonate (DEPC) and poly(vinyl alcohol) (PVA) (MW, 31,000–50 000 g/mol) were purchased from Sigma-Aldrich (St. Louis, MO, USA). Spectra/Por Dialysis Membrane Standard RC Tubin 12–14 kDa was acquired from Spectrum Laboratories Incorporation (CA). Quant-iT RiboGreen RNA Reagent and Tris–EDTA buffer (10 mM Tris, 1 mM EDTA, pH 8.0) (TE Buffer) were obtained from Molecular Probes, Invitrogen (Paisley, UK).

### Synthesis of Empty and Loaded LNPs

Empty and loaded LNPs were produced using a coflow glass-capillary microfluidic device already optimized by Liu et al.^39^ Briefly, lipids (cKK-E12, DSPC, cholesterol, and DSPE-PEG at the molar ratio of 50:10:38.5:1.5) were dissolved in ethanol (inner phase), and 1% PVA (w/v) was dissolved in DEPC-treated MQ-water (outer phase). To load the RNAs, siRNA, mRNA, or siRNA + mRNA were dissolved in the aqueous phase. After optimization (SI), the flow rates selected were 1 mL/min for the inner phase and 20 mL/min for the outer phase. The empty, single-loaded, and dual-loaded NPs named as LNPs, siRNA LNPs, mRNA LNPs, and dual-loaded LNPs were dialyzed overnight against an excess of Milli-Q water.

### Size, PDI, Zeta Potential Measurements, and Colloidal Stability of LNPs

Size, polydispersity index (PDI), and zeta-potential were characterized using dynamic light scattering (DLS) and electrophoretic light scattering (ELS) using a Zetasizer Nano Instrument (Malvern Instrument Ltd., UK) as previously described.^36^ The colloidal stability of LNPs was evaluated by incubating LNPs in the cell medium used for human tenocytes and in a solution of 1× phosphate buffer saline (cytiva HyClone, Finland), as previously described.^37^

### NTA Analysis

Particle concentration and size was measured with the ZetaView PMX-120 NTA Instruments (Particle Metrix GmbH, Ammersee, Germany) equipped with a Z NTA cell assembly, a blue (488 nm, 40 mW) laser, and a CMOS camera with 640 × 480-pixel resolution. Samples were diluted in a total volume of 1 mL of particle-free ultrapure Milli-Q water to obtain 50–200 particles per frame. Videos in NTA mode were recorded at 11 positions across the measurement chamber in 2 s increments at 30 FPS framerate with a camera shutter speed at 100 s^–1^ and sensitivity at 85. Temperature was controlled at 22 °C for NTA. Videos were processed and outliers (>10% CV) were removed using the Grubbs method with the built-in ZetaView software (version 8.05.12 SP2). Particles between 10 and 1000 nm in diameter with a minimum trace length of 15 frames and a minimum brightness of 20 were included in the analysis. The dilution factor for empty LNPs and dual-loaded LNPs were 10,000 and 5,000, respectively.

### Cryo-EM

LNPs resuspended in DEPC-treated Milli-Q water were vitrified on glow-discharged electron microscopy grids, Quantifoil holey carbon R1.2/1.3 Cu 300 mesh, using a Leica EM GP plunger at 80% humidity and 1.5 s blotting time using front blotting. Cryo-EM grid screening and data collection were performed at the cryo-EM facility at the University of Helsinki in Finland using a ThermoFisher Scientific Talos Arctica operating at 200 kV and equipped with a Falcon 3 direct electron detector operating in linear mode. Images were collected at 57 and 120 k× magnification.

### Quantification of RNAs from LNPs

The amount of RNAs in the LNPs was determined by using the Quant-iT RiboGreen RNA Reagent, as previously described.^36^

The encapsulation efficiency (EE) was calculated using eq 1:
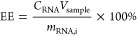
1where *C*_RNA_ is the concentration of RNA quantified (in ng mL^–1^), *V*_sample_ is the volume of sample (in mL), and *m*_RNA,i_ is the initial amount of siRNA added (in ng).

### Materials for Cell Culture Studies

RAW 264.7 cells were obtained from the American Type Culture Collection (USA). eGFP-expressing RAW 264.7 cells were purchased from Cellomics Technology (Rockville, Maryland, USA). Human primary tenocytes from the *human flexor digitorum profundus* were used to assess the in vitro compatibility, cell uptake, and *in vitro* therapeutic efficacy of the developed LNPs and were used at passages #2 to #7 in all the *in vitro* studies. The isolation of human tenocytes is described in the Supporting Information. Dulbecco’s modified Eagle medium (DMEM F-12, Gibco), supplemented with 10% of fetal bovinum serum (FBS, Gibco USA), 1% of penicillin and streptomycin (PEST, Gibco, USA), and 200 mM ascorbic acid (Sigma-Aldrich, USA) was used to grow tenocytes in an incubator (ESCO Celculture CO_2_ incubator, ESCO Scientific) at 37 °C, 5% of CO_2_, and 95% relative humidity. Roswell Park Memorial Institute (RPMI, Life Technologies Gibco, USA), supplemented with 10% FBS and 1% of penicillin and streptomycin solution, was used to grow RAW 264.7, in an incubator (BB 16 gas incubator, Heraeus Instruments GmbH) at 37 °C, 5% of CO_2_, and 95% relative humidity. The same cell media were used to grow eGFP-expressing RAW 264.7, without the 1% of penicillin and streptomycin supplement.

### Cytotoxicity Studies

The cytocompatibility of the nanosystems was assessed in human tenocytes. The human primary tenocytes were isolated, as described in the Supporting Information, from a 25 year-old patient after surgery of the *flexor digitorum profundus* tendon. Human primary tenocytes were seeded in a 96-well plate (Corning, USA) at density of 10,000 cells per well and were left to attach overnight. Suspensions of empty LNPs, siRNA-loaded LNPs, mRNA-loaded LNPs, dual-loaded LNPs, and mix LNPs with cell medium were prepared at the final LNPs concentration of 25, 50, 100, 250, and 500 μg/mL, and cells with complete medium and Triton X-100 (Merck Millipore, Darmstadt, Germany) were used as negative and positive controls. When mixed LNPs were used at the concentration of 50 μg/mL, 50 μg/mL of siRNA LNPs and 50 μg/mL of mRNA LNPs were mixed and added into the wells: this was done for each concentration. After 24 and 48 h of incubation (37 °C, 5% of CO_2_, and 95% relative humidity), cells were washed twice with Hank’s Balanced Salt Solution-(N-[2-hydroxyethyl]piperazine-*N*′-[2-ethanesulfonic acid]) (HBSS-HEPES, pH 7.4) and then 100 μL of HBSS-HEPES and CellTiter-Glo (1:1) were added to the cells. Finally, the luminescence was read with the Varioskan multimodal plate reader at 750 nm. HBSS and HEPES were purchased from cytiva HyClone, Finland and Sigma, USA, respectively.

### Transfection Efficiency of eGFP siRNA and eGFP mRNA LNPs

eGFP-expressing RAW 264.7 and RAW 264.7 cells were employed for preliminary *in vitro* transfection efficiency studies. The cells were seeded in 24-well plates at a density of 50,000 cells per well and left attached overnight. Suspensions of eGFP-siRNA and eGFP-mRNA LNPs in cell media were added at different concentrations (100 and 200 μg/mL) in eGFP-expressing RAW 264.7 and RAW 264.7, respectively. Lipofectamine RNAiMAX was used as a positive control at the concentration suggested by the manufacturer. Cells were processed as previously described.^36^ The transfection efficiency was evaluated by measuring the percentage of eGFP+ cells by BD Accuri C6 Plus (BD, USA) flow cytometry.

### Quantitative Uptake of FITC-Labeled LNPs on Human Tenocytes

Human primary tenocytes were seeded into a 12-well plate (Corning, USA) at a density of 100,000 cells per well and allowed to attach at 37 °C overnight. Cells were processed as previously described.^37^ The results were analyzed with FlowJo software v.10 (Tree Star, Inc., USA).

### Qualitative Uptake of FITC-Labeled LNPs in Human Tenocytes

Qualitative uptake of LNPs was studied by confocal imaging in human Tenocytes. Cells were seeded at a concentration of 30,000 in an 8-well chamber (ThermoFisher Scientific, USA) and allowed to attach overnight. Cells were processed as previously described.^36^ The images were captured with a Leica TCS SP8 STED 3× CW 3D Inverted Microscope (Leica Microsystems, Germany), using a 63× water objective, and analyzed with Leica AS software (Leica Microsystems, Germany).

### Real-Time Quantitative Polymerase Chain Reaction (RT-qPCR)

The antifibrotic effect of *SMAD3* siRNA and the pro-regenerative activity of COL1A1 mRNA LNPs were evaluated at the gene level by RT-qPCR. Human primary tenocytes were seeded in a 24-well plate (Corning, USA) at density of 50,000 cells per well and allowed to attach overnight. Empty LNPs (100 μg/mL), siRNA LNPs (LNPs concentration of 100 μg/mL corresponding to siRNA of 0.3 μg/mL), mRNA LNPs (LNPs concentration of 100 μg/mL corresponding to mRNA concentration of 0.3 μg/mL), dual-loaded LNPs (LNPs concentration of 100 μg/mL corresponding to siRNA and mRNA concentrations of 0.3 μg/mL, respectively), mixed LNPs (100 μg/mL of siRNA LNPs + 100 μg/mL of mRNA LNPs equivalent to siRNA and mRNA concentrations of 0.3 μg/mL, respectively), and the controls SMAD3 siRNA (0.3 μg/mL) and COL1A1 mRNA (0.3 μg/mL) were added to the cells for 48 h. Tenocytes with DMEM-F12 were used as a negative control. The isolation of RNA, the cDNA synthesis, and the amplification were performed as previously described.^37^ The probes used in the assay were from Thermo Fisher Scientific and predesigned: *18S* (4333760T), human Transforming Growth Factor-b (*TGFB1*, Hs00998133_m1), collagen type III alpha 1 chain (*COL3A1*, Hs00943809_m1), bone morphogenetic protein 2 (*BMP2*, Hs00154192_m1), and SMAD family member 3 (SMAD3 Hs00969210_m1). The ΔΔ*CT* of each sample was quantified, and the results were normalized to the housekeeping gene 18S.

### Quantification of Collagen I Production

Human tenocytes were seeded in a 96-well plate at a density of 7,500 cells per well and allowed to attach overnight. TGF- β1 (20 ng/mL) was added as positive control, empty LNPs (100 μg/mL), mRNA LNPs (LNPs concentration of 100 μg/mL corresponding to mRNA concentration of 0.3 μg/mL), dual-loaded LNPs (LNPs concentration of 100 μg/mL corresponding to siRNA and mRNA concentrations of 0.3 μg/mL, respectively), mixed LNPs (100 μg/mL of siRNA LNPs + 100 μg/mL of mRNA LNPs equivalent to siRNA and mRNA concentrations of 0.3 μg/mL, respectively), and COL1A1 mRNA (0.3 μg mL^–1^) were added to the cells for 48 h. Untreated human tenocytes (only cells) were used as negative control. Cells were fixed with 4% PFA and permeabilized with 0.1% Triton X-100 and blocking was done by incubating with 1% BSA for 30 min. A 50 μL per well volume of COL1A1 primary antibody (Abcam, USA, ab138496) was added at a 1:500 dilution and incubated overnight at 4 °C. Cells were washed three times with 1× PBS and the cells were incubated for 1 h with the secondary antibody Alexa Fluor 568 (Abcam, USA, ab138492) used at a 1:1000 dilution. Cells were washed three times with 1× PBS, and 50 μL of 1× PBS was added per well for imaging. Imaging was performed using a MolecularDevices Nano Instrument. 32 images were taken per well, and the images were processed using the MetaXpress software for quantitative determination of the collagen I production in each well. Briefly, standard global Otsu thresholding was used to segment the cells and the morphology and the Texas Red intensity values were calculated. Some regions with, for example, air bubbles and too high variation were left out of the segmentation profile to keep the data consistent.

### Intracellular Staining for SMAD3 Quantification

Human tenocytes were seeded in 24-well plates at a density of 75,000 cells per well and were allowed to attach overnight. Then, TGF-β1 (20 ng/mL), siRNA-LNPs (LNPs concentration of 100 μg/mL corresponding to mRNA concentration of 0.3 μg/mL), dual-loaded LNPs (LNPs concentration of 100 μg/mL corresponding to siRNA and mRNA concentrations of 0.3 μg mL^–1^, respectively), and mixed LNPs (100 μg/mL of siRNA-LNPs + 100 μg/mL of mRNA-LNPs equivalent to siRNA and mRNA concentrations of 0.3 μg/mL, respectively) were added for 48h. TGF- β1 treated cells were used as control of SMAD3 overexpression. After 48 h incubation with the LNPs, intracellular staining was performed using a PE-labeled anti-SMAD3 antibody (562586, BD Biosciences). Briefly, cells were detached with trypsin and were fixed with IC Fixation buffer (Paisley, UK) upon incubation for 1 h. Then, cells were centrifuged at 400*g* for 5 min, and the supernatant was discarded and 1× permeabilization buffer (Paisley, UK). Cells were washed again, and the pellet was resuspended in a 1:1 mixture of the permeabilization buffer and 5 μL of the antibody. The antibody was incubated for 40 min at room temperature and protected from light. Finally, cells were washed two times with 1× PBS and resuspended in 400 μL of PBS for flow cytometry analysis with a BD LSRFortessa Cells Analyzer (BD Bioscience, USA).

### Ethical Permissions

Patient’s recruitment, participation, and sample collection were obtained after receipt of a signed informed consent, approved by the Helsinki and Uusimaa Hospital District ethics committee (HUS/2785/2020) and by the institutional review board (HUS/234/2020).

### Statistical Analysis

The statistical analysis was performed in GraphPad Prism 10 (GraphPad Software, Inc., La Jolla, CA, USA). A detailed description of the statistical methods used to analyze the data is reported in the figure legend. In general, an ordinary one-way ANOVA followed by a Dunnett post hoc test and a pair Student’s *t*-test were used for the statistical analyses of the different studies.

## Supporting Information Available

Additional table of the optimization of LNPs, additional results and discussion of the transfection efficiency study, and additional figures of the cell uptake (PDF)
