# Supplementary material for: Antifibrotic and Pro-regenerative Effects of SMAD3 siRNA and Collagen I mRNA-Loaded Lipid Nanoparticles in Human Tenocytes
Source: ACS Appl Nano Mater. 2024 Jul 18;7(15):17736–47. doi: 10.1021/acsanm.4c02996 (PMC11320386; doi:10.1021/acsanm.4c02996)
Supplement: Supplementary file 1 — an4c02996_si_001.pdf [file an4c02996_si_001.pdf]

SUPPORTING INFORMATION

Anti-fibrotic and Pro-regenerative Effects of SMAD3 siRNA and Collagen I mRNA-loaded Lipid Nanoparticles in Human Tenocytes

Sandra López-Cerdá[1,§,], Giuseppina Molinaro[1,§,*], Rubén Pareja Tello[1], Alexandra Correia[1], Eero Waris[2], Jouni Hirvonen[1], Goncalo Barreto[3,4,5], Hélder A. Santos[1,6,*]*

[1] Drug Research Program, Division of Pharmaceutical Chemistry and Technology, Faculty of Pharmacy, FI-00014, Helsinki, Finland

[2] Department of Hand Surgery, University of Helsinki and Helsinki University Hospital, Helsinki, Finland

[3] Translational Immunology Research Program, Faculty of Medicine, University of Helsinki, PL 4 (Yliopistonkatu 3), 00014, Helsinki, Finland

[4] Medical Ultrasonics Laboratory (MEDUSA), Department of Neuroscience and Biomedical Engineering, Aalto University, 02150, Espoo, Finland

[5] Orton Orthopedic Hospital, Tenholantie 10, 00280 Helsinki, Finland

[6] Department of Biomedical Engineering, The Personalized Medicine Research Institute (PRECISION) the Personalized Medicine Research Institute (PRECISION), University Medical Center Groningen (UMCG), University of Groningen, Ant. Deusinglaan 1, 9713 AV Groningen, The Netherlands

*Corresponding authors: h.a.santos@umcg.nl
sandra.lopezcerda@helsinki.fi
giuseppina.molinaro@helsinki.fi

[§] S.L.C. and G.M. contributed equally to the paper.

Results and Discussion

Table S1. Optimization of the flow rate ratio of the aqueous and organic phases and of the concentration of lipids in the organic phase for the development of a co-flow single-step microfluidics production method. The flow rate ratio of 1-20 ml/min and the concentration of organic phase of 5 mg/mL were selected as optimal.

Flow rate aqueous phase	Flow rate organic phase	Concentration organic phase	Size (nm)	PDI
25 ml/hr	25 ml/hr	5 mg/ml	Aggregation, too less formulation obtained	
10 ml/hr	90 ml/hr	10 mg/ml	555	0.6
3 ml/hr	90 ml/hr	5 mg/ml	480	0.5
1 ml/hr	25 ml/hr	5 mg/ml	600	0.5
1 ml/min	1 ml/min	5 mg/ml	Aggregation	
1 ml/min	10 ml/min	5 mg/ml	489	0.5
1 ml/min	15 ml/min	10 mg/ml	315	0.4
**1 ml/min**	**20 ml/min**	**5 mg/mL**	**151**	**0.2**

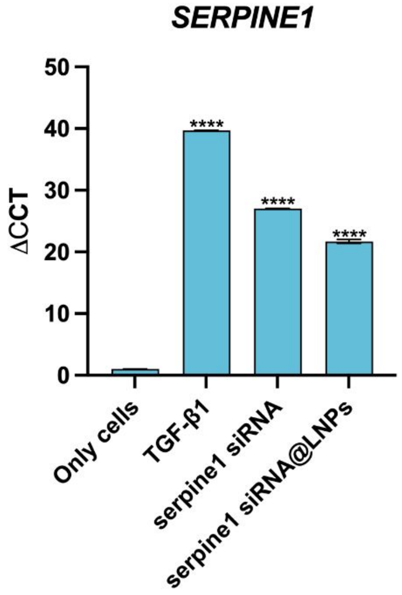

Figure S1. Transfection efficiency of siRNA LNPs loaded with the the irrelevant serpine1 siRNA. The expression of serpine1 was evaluated in human tenocytes by RT-qPCR. Results are

represented as fold increase values compared to the only cells control ± s.d. ($n \geq 3$). An ordinary one-way ANOVA followed by a Dunnett post-hoc test was used for the statistical analysis. The significance levels of the differences were set at the probabilities of $****p < 0.0001$ for comparison with only cells.

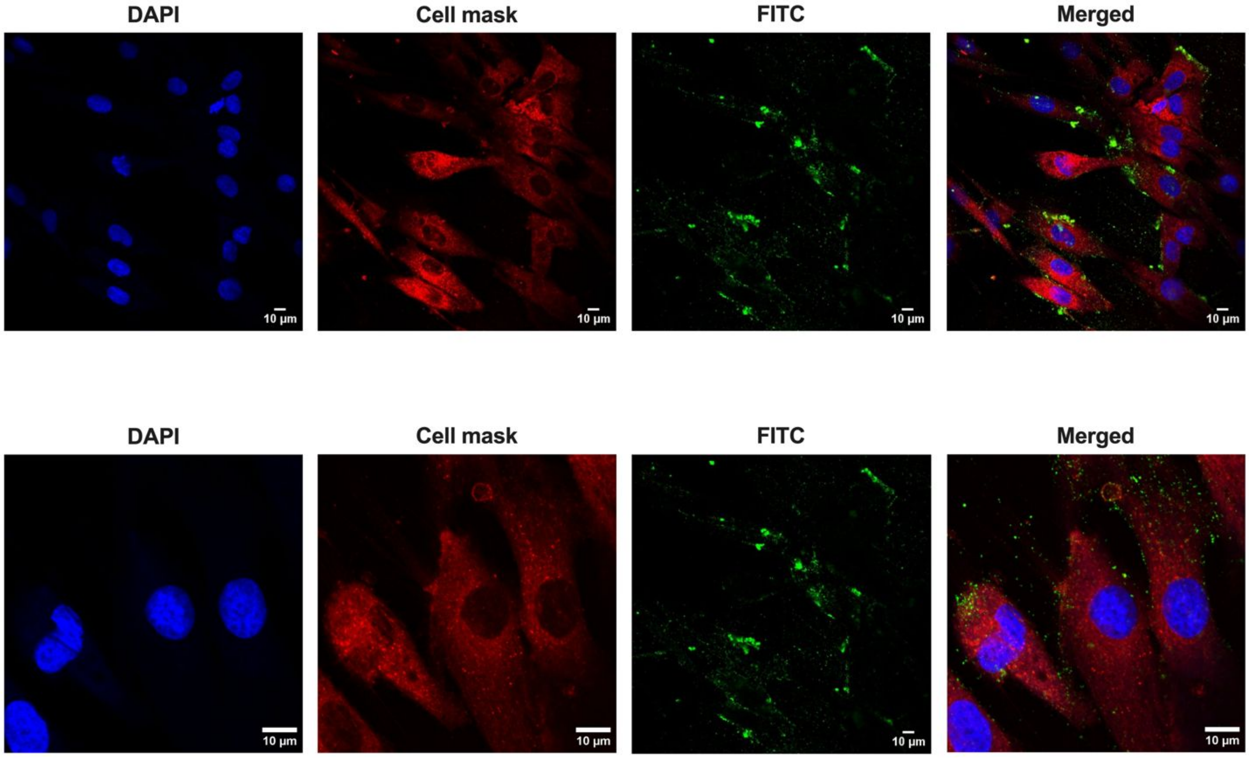

Figure S2. Qualitative uptake studies of LNPs in human tenocytes. The cell uptake was evaluated by confocal fluorescence microscopy after incubation with the FITC-labelled LNPs for 6h at 37 °C. The LNPs were visualized in FITC (green channel), while cells were stained with DAPI (nuclei, blue channel) and CellMask (cell membrane, red channel). Scale bars are shown in each image.
